# Association of Use of Omega-3 Polyunsaturated Fatty Acids With Changes in Severity of Anxiety Symptoms: A Systematic Review and Meta-analysis

**DOI:** 10.1001/jamanetworkopen.2018.2327

**Published:** 2018-09-14

**Authors:** Kuan-Pin Su, Ping-Tao Tseng, Pao-Yen Lin, Ryo Okubo, Tien-Yu Chen, Yen-Wen Chen, Yutaka J. Matsuoka

**Affiliations:** 1 Department of Psychiatry, China Medical University Hospital, Taichung, Taiwan; 2 Mind-Body Interface Laboratory (MBI-Lab), China Medical University Hospital, Taichung, Taiwan; 3 College of Medicine, China Medical University, Taichung, Taiwan; 4 WinShine Clinics in Specialty of Psychiatry, Kaohsiung City, Taiwan; 5 Department of Psychiatry, Kaohsiung Chang Gung Memorial Hospital, Kaohsiung, Taiwan; 6 Chang Gung University College of Medicine, Kaohsiung, Taiwan; 7 Institute for Translational Research in Biomedical Sciences, Kaohsiung Chang Gung Memorial Hospital, Kaohsiung, Taiwan; 8 Division of Health Care Research, Center for Public Health Sciences, National Cancer Center Japan, Tokyo, Japan; 9 Department of Psychiatry, Tri-Service General Hospital, Taipei, Taiwan; 10 School of Medicine, National Defense Medical Center, Taipei, Taiwan; 11 Institute of Brain Science, National Yang-Ming University, Taipei, Taiwan; 12 Prospect Clinic for Otorhinolaryngology & Neurology, Kaohsiung, Taiwan

## Abstract

**Importance:**

No systematic review or meta-analysis has assessed the efficacy of omega-3 polyunsaturated fatty acids (PUFAs) for anxiety.

**Objective:**

To evaluate the association of anxiety symptoms with omega-3 PUFA treatment compared with controls in varied populations.

**Data Sources:**

PubMed, Embase, ProQuest, ScienceDirect, Cochrane Library, ClinicalKey, Web of Science, and ClinicalTrials.gov databases were searched up to March 4, 2018.

**Study Selection:**

A search was performed of clinical trials assessing the anxiolytic effect of omega-3 PUFAs in humans, in either placebo-controlled or non–placebo-controlled designs. Of 104 selected articles, 19 entered the final data extraction stage.

**Data Extraction and Measures:**

Two authors independently extracted the data according to a predetermined list of interests. A random-effects model meta-analysis was performed and this study was conducted based on Preferred Reporting Items for Systematic Reviews and Meta-analyses guidelines.

**Main Outcomes and Measures:**

Changes in the severity of anxiety symptoms after omega-3 PUFA treatment.

**Results:**

In total, 1203 participants with omega-3 PUFA treatment (mean age, 43.7 years; mean female proportion, 55.0%; mean omega-3 PUFA dosage, 1605.7 mg/d) and 1037 participants without omega-3 PUFA treatment (mean age, 40.6 years; mean female proportion, 55.0%) showed an association between clinical anxiety symptoms among participants with omega-3 PUFA treatment compared with control arms (Hedges *g*, 0.374; 95% CI, 0.081-0.666; *P* = .01). Subgroup analysis showed that the association of treatment with reduced anxiety symptoms was significantly greater in subgroups with specific clinical diagnoses than in subgroups without clinical conditions. The anxiolytic effect of omega-3 PUFAs was significantly better than that of controls only in subgroups with a higher dosage (at least 2000 mg/d) and not in subgroups with a lower dosage (<2000 mg/d).

**Conclusions and Relevance:**

This review indicates that omega-3 PUFAs might help to reduce the symptoms of clinical anxiety. Further well-designed studies are needed in populations in whom anxiety is the main symptom.

## Key Points

**Question:**

Is omega-3 polyunsaturated fatty acid treatment associated with an improvement in anxiety symptoms?

**Findings:**

In this systematic review and meta-analysis of 19 clinical trials including 2240 participants from 11 countries, improvement in anxiety symptoms was associated with omega-3 polyunsaturated fatty acid treatment compared with controls in both placebo-controlled and non–placebo-controlled trials. The anxiolytic effects of omega-3 polyunsaturated fatty acids were also stronger in participants with clinical conditions than in subclinical populations.

**Meaning:**

Omega-3 polyunsaturated fatty acid treatment for anxiety might be effective in clinical settings.

## Teaser

This systematic review and meta-analysis of 19 clinical trials evaluates whether improvement in anxiety symptoms is associated with anxiolytic effects of omega-3 polyunsaturated fatty acid (PUFA) treatment compared with control treatments in both placebo-controlled and non–placebo-controlled trials.

## Introduction

Anxiety, the most commonly experienced psychiatric symptom, is a psychological state derived from inappropriate or exaggerated fear leading to distress or impairment. The lifetime prevalence of any anxiety disorder is reported to be approximately 1 in 3.^1^ Anxiety is often comorbid with depressive disorders^2^ and is associated with lower health-related quality of life^3^ and increased risk of all-cause mortality.^4^ Treatment options include psychological treatments, such as cognitive-behavioral therapy and pharmacological treatments, mainly with selective serotonin reuptake inhibitors.^5^ Individuals with anxiety and related disorders tend to be more concerned about the potential adverse effects of pharmacological treatments (eg, sedation or drug dependence) and may be reluctant to engage in psychological treatments that can be time-consuming and costly, as well as sometimes limited in availability.^6^ Thus, evidence-based and safer treatments are required, especially for anxious patients with comorbid medical conditions.

Omega-3 polyunsaturated fatty acids (PUFAs), such as eicosapentaenoic acid (EPA) and docosahexaenoic acid (DHA), are essential nutrients that have potential preventive and therapeutic effects on psychiatric disorders, such as anxiety and depression,^7,8,9,10,11,12,13,14,15^ as well as comorbid depression and anxiety in physically ill patients,^16,17,18,19^ patients with coronary heart disease,^20,21^ and pregnant women.^22,23^ Preclinical data support the effectiveness of omega-3 PUFAs as treatment for anxiety disorders. Song et al^24,25^ found that an EPA-rich diet could reduce the development of anxiety-like behaviors in rats as well as normalize dopamine levels in the ventral striatum. In addition, Yamada et al^26^ showed that a high dietary omega-3 to omega-6 PUFA ratio reduced contextual fear behaviors in mice and that these effects were abolished by a cannabinoid CB1 receptor antagonist.

A number of trials have found that omega-3 PUFAs might reduce anxiety under serious stressful situations. Case-controlled studies have shown low peripheral omega-3 PUFA levels in patients with anxiety disorders.^27,28,29,30,31^ A cohort study found that high serum EPA levels were associated with protection against posttraumatic stress disorder.^32^ In studies of therapeutic interventions, while a randomized clinical trial of adjunctive EPA treatment in patients with obsessive-compulsive disorder revealed that EPA augmentation had no beneficial effect on symptoms of anxiety, depression, or obsessive-compulsiveness,^33^ a randomized clinical trial involving participants with substance abuse showed that EPA and DHA administration was accompanied by significant decreases in anger and anxiety scores compared with placebo.^34^ In addition, a randomized clinical trial found that omega-3 PUFAs had additional effects on decreasing depressive and anxiety symptoms in patients with acute myocardial infarction,^35^ and a randomized clinical trial demonstrated that omega-3 PUFAs could reduce inflammation and anxiety among healthy young adults facing a stressful major examination.^36^ Despite the largely positive findings of these trials, the clinical application of the findings is unfortunately limited by their small sample sizes.

We hypothesized that omega-3 PUFAs might have anxiolytic effects in patients with significant anxiety- and fear-related symptoms. However, there have been no systematic reviews of this topic to date. Thus, we examined the anxiolytic effects of omega-3 PUFAs in participants with elevated anxiety symptoms in the results of clinical trials to determine the overall efficacy of omega-3 PUFAs for anxiety symptoms irrespective of diagnosis.

## Methods

This systematic review followed the Preferred Reporting Items for Systematic Reviews and Meta-analyses (PRISMA) reporting guidelines.^37^ The study protocol adhered to the requirements of the institutional review board of Tri-Service General Hospital.

### Literature Search and Screening

Two psychiatrists (P.-T.T. and T.-Y.C.) separately performed a systematic literature search of the PubMed, Embase, ProQuest, ScienceDirect, Cochrane Library, ClinicalKey, Web of Science, and ClinicalTrials.gov databases to March 4, 2018. Because we presumed some clinical trials would use investigating scales for some other mood symptoms but also contain symptoms of anxiety, we tried to use some nonspecific medical subject heading terms to include those clinical trials. Therefore, we used the following keywords: omega-3, eicosapentaenoic acid, EPA, DHA, or docosahexaenoic acid; and anxiety, anxiety disorder, generalized anxiety disorder, agoraphobia, panic disorder, or posttraumatic stress disorder. After removing duplicate studies, the same 2 authors screened the search results according to the title and abstract to evaluate eligibility. List of potentially relevant studies were generated for a full-text review. Any inconsistencies were discussed with a third author to achieve final consensus. To expand the list of potentially eligible articles, we performed a manual search of the reference lists of review articles in this area.^12,38,39^

**Figure 1.**
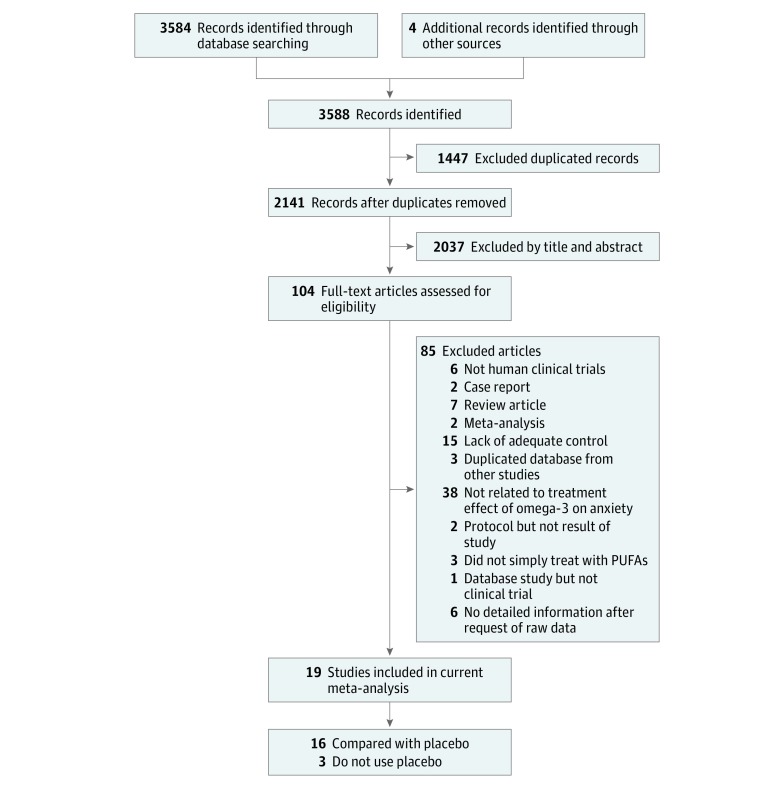
Flowchart of the Selection Strategy and Inclusion and Exclusion Criteria for This Meta-analysis PUFAs indicates polyunsaturated fatty acids.

Because of the preliminary state of knowledge on the effects of omega-3 PUFA treatment on anxiety, we decided to include as many studies as possible and not to set further limitations on specific characteristics, such as length of study, diagnosis, omega-3 PUFA dosage, omega-3 PUFA preparation (EPA to DHA ratio), rated anxiety coding scale, or type of control. Therefore, we chose to make the inclusion criteria as broad as possible to avoid missing any potentially eligible studies. The inclusion criteria included clinical trials in humans (randomized or nonrandomized), studies investigating the effects of omega-3 PUFA treatment on anxiety symptoms, and formal published articles in peer-reviewed journals. The clinical trials could be placebo controlled or non–placebo controlled. The target participants could include healthy volunteers, patients with psychiatric illness, and patients with physical illnesses other than psychiatric illnesses. The exclusion criteria included case reports or series, animal studies or review articles, and studies not investigating the effects of omega-3 PUFA treatment on anxiety symptoms. We did not set any language limitation to increase the number of eligible articles. Figure 1 shows the literature search and screening protocol.

### Meta-analysis and Data Extraction and Input

Due to the anticipated heterogeneity, a random-effects meta-analysis was chosen rather than a fixed-effects meta-analysis because random-effects modeling is more stringent and incorporates an among-study variance in the calculations. The entire meta-analysis procedure was performed on the platform of Comprehensive Meta-analysis statistical software, version 3 (Biostat). Under the preliminary assumption that the scales for anxiety symptoms are heterogeneous among the recruited studies, we chose Hedges *g* and 95% confidence intervals to combine the effect sizes, in accordance with the manual of the Comprehensive Meta-analysis statistical software, version 3. Regarding the interpretation of effect sizes, we defined Hedges *g* values 0 or higher as a better association of treatment with reduced anxiety symptoms of omega-3 PUFAs than in controls. For each analysis, a 2-tailed *P* value less than .05 was considered to indicate statistical significance. When more than 1 anxiety scale was used in a study, we chose the one with the most informative data (ie, mean and standard deviation [SD] before and after treatment). We entered the primary outcome provided in the included articles or obtained from the original authors. As for the variance imputation, we mainly chose the mean and SD before and after treatment. Later, we entered the mean and SD and calculated the effect sizes based on the software option, standardized by post score SD. In the case of studies with 2 active treatment arms, we merged the 2 active treatment arms into 1 group. If these 2 active treatment arms belonged to different subgroups (ie, different PUFA dosage subgroups), we kept them separate. Regarding the numbers of participants counted, we chose intention-to-treat as our priority. If there were insufficient data in the intention to treat group (ie, some studies only provided the changes in anxiety severity in those participants completing trials), we chose instead the per-protocol numbers of participants.

The quality of the included clinical trials were assessed using the Jadad score,^40^ which was designed to evaluate the risk of bias in interventional trials in 3 specific domains: randomization, blindness, and cohort follow-up.

The primary outcome was analyzed by changes in anxiety symptoms in patients receiving omega-3 PUFA treatment compared with those not receiving omega-3 PUFA treatment.

### Heterogeneity, Publication Bias, and Sensitivity Testing

Heterogeneity was examined using the Q statistic and the corresponding *P* values,^41^ and the *I*^2^ statistic was used to evaluate the proportion of variation resulting from among-study differences. Any possible publication bias was detected with both funnel plots and Egger regression in the main part of the meta-analysis.^42^ By using Duval and Tweedie’s trim-and-fill test, we adjusted the effect sizes for potential publication bias if there was evidence of publication bias detected by this test in the Comprehensive Meta-analysis statistical software, version 3.^43^ To investigate the potential confounding effects of any outliers within the recruited studies, sensitivity testing was conducted with the 1-study removal method to detect the potential outliers.^44^

### Metaregression and Subgroup Meta-analysis

To exclude the possible confounding effects of clinical variables on the Hedges *g*, metaregression analysis was conducted with an unrestricted maximum likelihood random-effects model of single variables when there were more than 10 data sets available. Specifically, the clinical variables of interest included mean age, female proportion, sample size, mean body mass index, daily omega-3 PUFA dosage, EPA to DHA ratio, treatment duration, dropout rate, and others. In addition, a subgroup meta-analysis was conducted to investigate potential sources of heterogeneity, specifically, a further subgroup meta-analysis focused on those trials that were placebo controlled or non–placebo controlled. To more clearly uncover the differences in the meta-analysis results among the recruited studies, a further subgroup meta-analysis was performed according to the presence of a specific clinical diagnosis or no specific clinical condition, mean omega-3 PUFA daily dosage, and mean age. In addition, in a previous study, the EPA percentage (ie, ≥60%) in the PUFA regimens had different effects on depression treatment.^9^ Therefore, we also arranged the subgroup meta-analysis based on the EPA percentage. Furthermore, we arranged subgroup meta-analysis procedures only when there were at least 3 data sets included.^45^ To investigate the potentially different estimated effect sizes between subgroups, we performed an interaction test and calculated the corresponding *P* values.^46^

## Results

### Characteristics of the Included Studies

After the initial screening process, a total of 104 articles were considered for full-text review (Figure 1; eFigure 1 in the Supplement); 85 were excluded according to the exclusion criteria (eAppendix in the Supplement), leaving 19 articles for analysis in this study (Table).^33,34,35,36,47,48,49,50,51,52,53,54,55,56,57,58,59,60,61^

**Table.** Characteristics of Recruited Studies

Source	Diagnosis	Comparison	Participants, No.	Anxiety Scale	Age, Mean (SD), y	Female, No. (%)	Omega-3 Dosage, mg/d	Dropout Rate, No./Total No.	Treatment Duration, wk	Country
Watanabe et al,^47^ 2018	Junior nurses work in hospital	Omega-3 PUFA Placebo	40 40	HADS-A	29.6 (9.1) 30.5 (7.8)	40 (100.0) 40 (100.0)	1800.0	0/40 3/40	13	Japan
Cornu et al,^48^ 2018	Children with ADHD	Omega-3 PUFA Placebo	80 82	Conners	10.2 (2.8) 9.7 (2.5)	19 (23.7) 16 (19.5)	600.0	3/80 1/82	12	France
Matsuoka et al,^49^ 2015	Severe accidental injury	Omega-3 PUFA Placebo	53 57	CAPS	38.1 (13.5) 40.9 (17.3)	9 (17.0) 11 (19.3)	2100.0	8/53 6/57	12	Japan
Bellino et al,^50^ 2014	Borderline personality disorders	Omega-3 PUFA + valproate Control + valproate	18 16	HAM-A	25.2 (6.4)	26 (76.5)	2000.0	5/23 4/20	12	Italy
Cohen et al,^51^ 2014	Generally healthy participants	Omega-3 PUFA Placebo	177 178	GAD-7	54.7 (3.7)	177 (100.0) 178 (100.0)	1800.0	4/177 5/178	12	United States
Pomponi et al,^52^ 2014	Parkinson disease	Omega-3 PUFA Placebo	12 12	HAM-A	64.0 (4.9) 64.0 (9.8)	5 (41.7) 6 (50.0)	2000.0	0/12 0/12	24	Italy
Widenhorn-Müller et al,^53^ 2014	Children with ADHD	Omega-3 PUFA Placebo	46 49	CBCL-A	8.9 (1.5) 8.9 (1.2)	11 (23.9) 10 (20.4)	720.0	7/55 6/55	16	Germany
Haberka et al,^35^ 2013	AMI	Omega-3 PUFA + AMI treatment Control + AMI treatment	26 26	STAI	56.4 59.6 (6.0)	3 (11.5) 4 (15.4)	1000.0	0/26 0/26	4	Poland
Nishi et al,^54^ 2013	Disaster-related trauma	Omega-3 PUFA + education Education	86 86	IES-R	37.9 (7.4) 37.4 (7.4)	24 (27.9) 23 (26.7)	2240.0	0/86 1/86	12.6	Japan
Sauder et al,^55^ 2013	Healthy, nonsmoking men and postmenopausal women with moderate hypertriglyceridemia	Omega-3 PUFA (3.4 g/d) Omega-3 PUFA (0.85 g/d) Placebo	26 26 26	STAI-state	44.0	3 (11.5)	3400.0 850.0	0/26 0/26 0/26	8	United States
Sohrabi et al,^56^ 2013	Women with premenstrual syndrome	Omega-3 PUFA Placebo	63 61	VASA	31.2 (6.5) 31.6 (8.4)	63 (100.0) 61 (100.0)	1000.0	7/70 8/69	12	Iran
Gabbay et al,^57^ 2012	Tourette syndrome	Omega-3 PUFA Placebo	17 16	C-YBOCS	11.9 (3.6) 10.6 (2.3)	3 (17.6) 3 (18.8)	4074.0	3/17 5/16	20	United States
Kiecolt-Glaser et al,^36^ 2011	Generally healthy participants	Omega-3 PUFA Placebo	34 34	BAI	23.9 (2.0) 23.4 (1.7)	16 (47.1) 14 (41.2)	2496.0	0/34 0/34	12	United States
Buydens-Branchey et al,^34^ 2008	Substance abuse	Omega-3 PUFA Placebo	11 11	POMS	NA	0 0	3000.0	0/11 0/11	12	United States
Freund-Levi et al,^58^ 2008	Alzheimer disease	Omega-3 PUFA Placebo	89 85	NPI	72.6 (9.0) 72.9 (8.6)	51 (57.3) 39 (45.9)	2320.0	12/103 14/101	24	Sweden
Rogers et al,^59^ 2008	Mild to severe depression	Omega-3 PUFA Placebo	109 109	DASS	38.0 (13.5) 38.2 (13.7)	85 (78.0) 83 (76.1)	2369.5	13/109 15/109	12	United Kingdom
van de Rest et al,^60^ 2008	Elderly volunteers	Omega-3 PUFA (1.8 g/d) Omega-3 PUFA (0.4 g/d) Placebo	96 100 106	HADS-A	69.9 (3.4) 69.5 (3.2) 70.1 (3.7)	43 (44.8) 45 (45.0) 47 (44.3)	1800.0 400.0	0/96 0/100 3/106	26	Netherlands
Yehuda et al,^61^ 2005	Undergraduate college students with test anxiety	Omega-3 PUFA Placebo	88 38	TAS	NA	NA	225.0	0/88 0/38	3	Israel
Fux et al,^33^ 2004	Obsessive-compulsive disorder	Omega-3 PUFA Placebo	6 5	YBOCS	33.5 (5)	8 (72.7)	2000.0	1/11	6	Israel

Abbreviations: ADHD, attention-deficit/hyperactivity disorder; AMI, acute myocardial infarction; BAI, Beck anxiety index; CAPS, clinician-administered posttraumatic stress disorder scale; CBCL-A, Child Behavior Checklist anxiety subscale; C-YBOCS, children’s Yale-Brown obsessive-compulsive scale; DASS, depression, anxiety, and stress scales; GAD-7, generalized anxiety disorder questionnaire; HADS-A, Hospital Anxiety and Depression Scale anxiety subscale; HAM-A, Hamilton anxiety rating scale; IES-R, impact of event scale-revised; NA, not available; NPI, Neuropsychiatric Inventory; POMS, profiles of mood states; PUFA, polyunsaturated fatty acid; STAI, state-trait anxiety inventory; TAS, test anxiety severity; VASA, visual analog scale of anxiety; YBOCS, Yale-Brown obsessive-compulsive scale.

In the 19 recruited studies,^33,34,35,36,47,48,49,50,51,52,53,54,55,56,57,58,59,60,61^ there were a total of 1203 participants with omega-3 PUFA treatment (mean age, 43.7 years; mean female proportion, 55.0%; mean omega-3 PUFA dosage, 1605.7 mg/d) and 1037 participants without omega-3 PUFA treatment (mean age, 40.6 years; mean female proportion, 55.0%).

Various scales were used in these studies to evaluate the target outcome of anxiety symptoms: the Yale-Brown Obsessive-Compulsive Scale, Profile of Mood States, State-Trait Anxiety Inventory, Hamilton Anxiety Rating Scale, Generalized Anxiety Disorder questionnaire, Depression, Anxiety, and Stress Scales, Clinician-Administered Posttraumatic Stress Disorder Scale, Beck Anxiety Inventory, visual analog scale of anxiety, Impact of Event Scale–Revised, Conners score anxiety subscale, Neuropsychiatric Inventory, test anxiety severity, Hospital Anxiety and Depression Scale anxiety subscale, and Child Behavior Checklist anxiety subscale. The psychiatric and physical health conditions of the recruited participants also varied widely: general population without specific clinical conditions,^36,47,51,55,60^ participants with acute myocardial infarction,^35^ borderline personality disorder,^2^ mild to severe depression,^59^ obsessive-compulsive disorder,^33^ severe accidental injury,^49^ participants who were traumatized by disaster,^54^ participants with substance abuse disorder,^34^ women with premenstrual syndrome,^56^ children with attention-deficit/hyperactivity disorder,^48,53^ Alzheimer disease,^58^ generally healthy undergraduate college students but with test anxiety,^61^ Parkinson disease,^52^ and participants with Tourette syndrome.^57^ Sixteen studies compared the effect of omega-3 PUFA treatment with that of the placebo^33,34,36,47,48,49,51,52,53,55,56,57,58,59,60,61^; the other 3 studies were non–placebo controlled trials.^35,50,54^ The mean (SD) Jadad score of the recruited studies was 3.8 (1.0) (eTable in the Supplement).

### Meta-analysis of Changes in Anxiety Symptoms in Patients Receiving and Not Receiving Omega-3 PUFA Treatment

**Figure 2.**
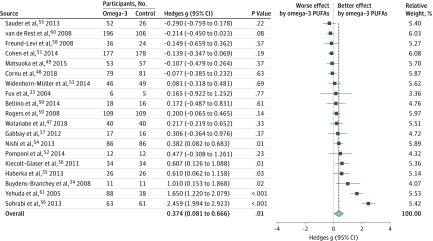
Meta-Analysis Forest Plot of the Association of Treatment With Reduced Anxiety Symptoms in Patients Receiving and Not Receiving omega-3 PUFAs There was a significant improvement in anxiety symptoms in patients receiving omega-3 PUFAs than in those not receiving omega-3 PUFAs (*k*, 19; Hedges *g*, 0.374; 95% CI, 0.081-0.666; *P* = .01).

In total, 19 articles with 19 data sets revealed the main results of the meta-analysis, namely that there was a significantly better association of treatment with reduced anxiety symptoms in patients receiving omega-3 PUFA treatment than in those not receiving it (*k*, 19; Hedges *g*, 0.374; 95% CI, 0.081-0.666; *P* = .01; Figure 2), with significant heterogeneity (Cochran Q, 178.820; *df*, 18; *I*^2^, 89.934%; *P* < .001) but no significant publication bias via Egger regression (*t*, 1.736; *df*, 17; *P* = .10) or inspection of the funnel plot (eFigure 2 in the Supplement). According to the trim-and-fill test, there was no need for adjustment for publication bias. The meta-analysis results remained significant after removal of any one of the included studies, which indicated that the significant results are not owing to any single study.

There was no significant association between the Hedges *g* and mean age (*k*, 17; *P* = .51), female proportion (*k*, 18; *P* = .32), mean omega-3 PUFA dosage (*k*, 19; *P* = .307), EPA to DHA ratio (*k*, 17; *P* = .86), dropout rate in the omega-3 PUFA group (*k*, 18; *P* = .71), duration of omega-3 PUFA treatment (*k*, 19; *P* = .14), Jadad score of randomization (*k*, 19; *P* = .10), Jadad score of blindness (*k*, 19; *P* = .57), or total Jadad score (*k*, 19; *P* = .18).

### Subgroup Meta-analysis When Focusing on Placebo-Controlled Trials or Non–Placebo-Controlled Trials

Among the 16 studies comparing the effect of omega-3 PUFA treatment with that of the placebo,^33,34,36,47,48,49,51,52,53,55,56,57,58,59,60,61^ the main results revealed a significantly greater association of treatment with reduced anxiety symptoms in patients receiving omega-3 PUFA treatment than in those not receiving it (*k*, 16; Hedges *g*, 0.372; 95% CI, 0.032-0.712; *P* = .03; eFigure 3 in the Supplement). The meta-analysis of the subgroup focusing on non–placebo-controlled trials also showed a significantly greater association of treatment with reduced anxiety symptoms in patients receiving omega-3 PUFA treatment than in those not receiving it (*k*, 3; Hedges *g*, 0.399; 95% CI, 0.154-0.643; *P* = .001).^35,50,54^

### Subgroup Meta-analysis When Focusing on Trials Recruiting Participants Without Specific Clinical Conditions or Trials Recruiting Participants With Specific Clinical Diagnoses

**Figure 3.**
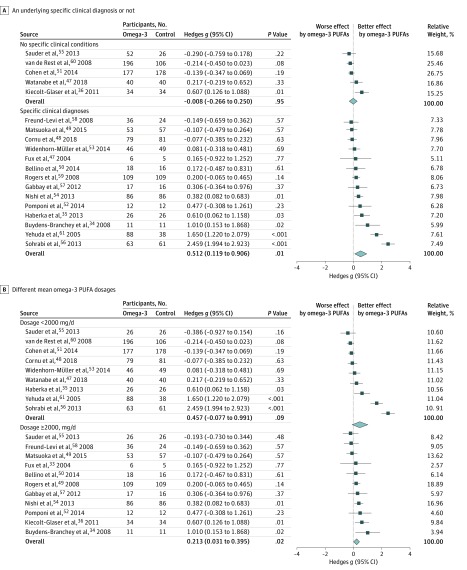
Forest Plot of Subgroup Meta-analysis A, Subgroup meta-analysis of the anxiolytic effect of omega-3 polyunsaturated fatty acids (PUFAs) based on an underlying specific clinical diagnosis or not. The anxiolytic effect of omega-3 PUFAs was not significant in the subgroup of participants without specific clinical conditions (*k*, 5; Hedges *g*, –0.008; 95% CI, –0.266 to 0.250; *P* = .95) but was significant in the subgroup of participants with specific clinical diagnoses (*k*, 14; Hedges *g*, 0.512; 95% CI, 0.119-0.906; *P* = .01). Furthermore, the association of treatment with reduced anxiety symptoms of omega-3 PUFAs were significantly stronger in subgroups with specific clinical diagnoses than in subgroups without specific clinical conditions (*P* = .03). B, Subgroup meta-analysis of the anxiolytic effect of omega-3 PUFAs based on different mean omega-3 PUFA dosages. The anxiolytic effect of omega-3 PUFAs was not significant in subgroups of mean omega-3 PUFA dosages less than 2000 mg/d (*k*, 9; Hedges *g*, 0.457; 95% CI, –0.077 to 0.991; *P* = .09) but was significant in the subgroup of mean omega-3 PUFA dosage of at least 2000 mg/d (*k*, 11; Hedges *g*, 0.213; 95% CI, 0.031-0.395; *P* = .02).

Five studies with 7 data sets recruited participants without specific clinical conditions.^36,47,51,55,60^ The main results revealed that there was no significant difference in the association of treatment with reduced anxiety symptoms between patients receiving omega-3 PUFA treatment and those not receiving it (*k*, 5; Hedges *g*, –0.008; 95% CI, –0.266 to 0.250; *P* = .95) (Figure 3A). Fourteen studies with 14 data sets recruited participants with specific clinical diagnoses.^33,34,35,48,49,50,52,53,54,56,57,58,59,61^ The main results revealed a significantly greater association of treatment with reduced anxiety symptoms in patients receiving omega-3 PUFA treatment than in those not receiving it (*k*, 14; Hedges *g*, 0.512; 95% CI, 0.119-0.906; *P* = .01) (Figure 3A). Furthermore, according to the interaction test, the association of omega-3 PUFA treatment with reduced anxiety symptoms was significantly stronger in subgroups with specific clinical diagnoses than in subgroups without specific clinical conditions (*P* = .03).

### Subgroup Meta-analysis When Focusing on Trials With Omega-3 PUFA Dosages of Less Than 2000 mg/d or at Least 2000 mg/d

Nine studies with 10 data sets used omega-3 PUFA dosages of less than 2000 mg/d.^35,47,48,51,53,55,56,60,61^ The main results revealed that there was no significant difference in the association of treatment with reduced anxiety symptoms between patients receiving omega-3 PUFA treatment and those not receiving it (*k*, 9; Hedges *g*, 0.457; 95% CI, –0.077 to 0.991; *P* = .09) (Figure 3B). Ten studies with 10 data sets used omega-3 PUFA dosages of at least 2000 mg/d.^33,34,36,49,50,52,54,55,57,58,59^ The main results revealed a significantly greater association of treatment with reduced anxiety symptoms in patients receiving omega-3 PUFA treatment than in those not receiving it (*k*, 11; Hedges *g*, 0.213; 95% CI, 0.031-0.395; *P* = .02) (Figure 3B). Furthermore, there was no significantly different estimated effect sizes between these 2 subgroups by the interaction test (*P* = .40).

### Subgroup Meta-analysis of Trials With an EPA Percentage Less Than 60% or an EPA Percentage of at Least 60%

**Figure 4.**
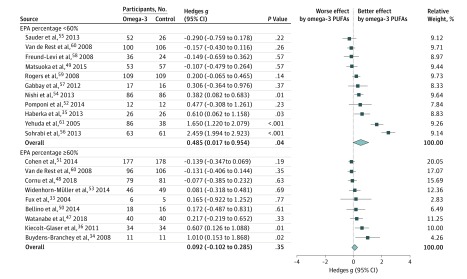
Subgroup Meta-analysis With Different Eicosapentaenoic Acid (EPA) Percentages Subgroup meta-analysis of the anxiolytic effects of omega-3 polyunsaturated fatty acids (PUFAs) based on different EPA percentages. The anxiolytic effects of omega-3 PUFAs were significant in the subgroup with an EPA percentage less than 60% (*k*, 11; Hedges g = 0.485; 95% CI, 0.017 to 0.954; *P* = .04) but not significant in the subgroups with an EPA percentage of at least 60% (*k*, 9; Hedges *g*, 0.092; 95% CI, –0.102 to 0.285; *P* = .35).

There was a significantly greater association of treatment with reduced anxiety symptoms in participants receiving omega-3 PUFAs than in those not receiving omega-3 PUFAs in the subgroup with an EPA percentage less than 60% (*k*, 11; Hedges *g*, 0.485; 95% CI, 0.017-0.954; *P* = .04; Figure 4)^35,49,52,54,55,56,57,58,59,60,61^ but no significant difference in the association of treatment with reduced anxiety symptoms between participants receiving omega-3 PUFAs and those not receiving omega-3 PUFAs in the subgroup with an EPA percentage of at least 60% (*k*, 9; Hedges *g*, 0.092; 95% CI, –0.102 to 0.285; *P* = .35) (Figure 4).^33,34,36,47,48,50,51,53,60^ There were no significantly different estimated effect sizes between these 2 subgroups by the interaction test (*P* = .13).

### Other Subgroup Meta-analyses of Changes in Anxiety Symptoms in Patients Receiving and Not Receiving Omega-3 PUFA Treatment

In addition, there was no significant difference in the association of treatment with reduced anxiety symptoms between participants receiving omega-3 PUFAs and those not receiving omega-3 PUFAs in the adolescent subgroup (aged <18 years) (*k*, 3; Hedges *g*, 0.020; 95% CI, –0.209 to 0.250; *P* = .86),^48,53,57^ in the adult subgroup (aged ≥18 years but <60 years) (*k*, 11; Hedges *g*, 0.388; 95% CI, –0.012 to 0.788; *P* = .06),^33,35,36,47,49,50,51,54,55,56,59^ or in the elderly subgroup (aged ≥60 years) (*k*, 3; Hedges *g*, –0.112; 95% CI, –0.406 to 0.181; *P* = .45).^52,58,60^ These insignificant results might be due to the smaller sample sizes in each subgroup.

## Discussion

To our knowledge, this is the first systematic review and meta-analysis to examine the anxiolytic effects of omega-3 PUFAs in individuals with anxiety symptoms. The overall findings revealed modest anxiolytic effects of omega-3 PUFAs in individuals with various neuropsychiatric or major physical illnesses. Although participants and diagnoses were heterogeneous, the main finding of this meta-analysis was that omega-3 PUFAs were associated with significant reduction in anxiety symptoms compared with controls; this effect persisted vs placebo controls. Furthermore, the association of treatment with reduced anxiety symptoms of omega-3 PUFA were significantly higher in subgroups with specific clinical diagnoses than in subgroups without clinical conditions.

Interestingly, the results are also consistent with our recent findings that somatic anxiety is associated with omega-3 PUFA deficits and the genetic risks of PUFA metabolic enzyme cytosolic phospholipase A2 in major depressive disorder^62,63^ and interferon α–induced neuropsychiatric syndrome.^63,64^ Brain membranes contain a high proportion of omega-3 PUFAs and their derivatives and most animal and human studies suggest that a lack of omega-3 PUFAs in the brain might induce various behavioral and neuropsychiatric disorders,^16,65,66,67,68,69,70^ including anxiety-related behaviors.^12,18,19,32,49,71^ Emerging evidence suggests that omega-3 PUFAs interfere with and possibly control several neurobiological processes, such as neurotransmitter systems, neuroplasticity, and inflammation,^12,72^ which is postulated to be the mechanism underlying anxiety and depression.

In our analysis, most of the included studies showed a positive Hedges *g* toward a beneficial effect of omega-3 PUFAs in anxiety reduction, although not all findings were statistically significant. However, after merging of these effect sizes from all of the included studies, the main result showed significant findings in our meta-analysis. Despite the significant heterogeneity, no significant publication bias was found among these 19 studies.

To evaluate the potential placebo effect, we made further subgrouping analyses. In the subgroups of studies using placebo controls, the omega-3 PUFAs still revealed a consistent positive anxiolytic association with anxiety symptoms. These phenomena meant that the anxiolytic effect of omega-3 PUFAs is probably not entirely owing to the placebo effect.

Further, according to subgroup results based on the presence of specific clinical diagnoses or not, the association of omega-3 PUFA treatment with reduced anxiety symptoms was significantly higher in subgroups with specific clinical diagnoses than in subgroups without clinical conditions. Among 6 studies included in a meta-analysis of the effect of omega-3 PUFAs on depressive symptoms, the analysis showed a nearly null effect of omega-3 PUFAs on depressive symptoms in healthy participants.^73^ Although the reason for the null effect of omega-3 PUFAs on anxiety and depressive symptoms remains unclear, certain pathophysiological conditions might be required for omega-3 PUFAs to exert an association of treatment with reduced anxiety symptoms.

Participants treated with a daily dose of 2000 mg or more of omega-3 PUFAs showed a significantly greater association of treatment with reduced anxiety symptoms. In addition, participants receiving supplements containing less than 60% EPA showed a significant association, but not those receiving supplements containing 60% or more EPA. The depression literature supports the clinical benefits of EPA-enriched formulations (≥60% or ≥50%) compared with placebo for the treatment of clinical depression.^9,13,73,74,75^ This opposite effect of EPA-enriched formations on anxiety and depression is intriguing and possibly linked to a distinct underlying mechanism of omega-3 PUFAs. Exploration of the effects of omega-3 PUFAs on anxiety symptoms is just beginning and studies assessing the dose response anxiolytic effects of omega-3 PUFAs have not yet been performed. Further phase 2 trials of anxiety symptoms among participants with neuropsychiatric illness or physical illness should aim to determine the optimal dose.

Although there was significant heterogeneity among the included studies (Cochran Q, 178.820; *df*, 18; *I*^2^, 89.934%; *P* < .001), the sensitivity test suggested that the main significant results of the meta-analysis would not change after removal of any of the included studies. However, through direct inspection of the forest plot, we detected the potential influence of some outliers, such as the studies by Sohrabi et al^56^ and Yehuda et al.^61^ These 2 studies evaluated anxiety symptoms with a visual analog scale of anxiety and test anxiety severity, which are seldom used in psychiatric research and lack a definite report to prove their equivalent sensitivity and specificity to some other frequently used anxiety rating scales, such as depression, anxiety, and stress scales or the Hamilton anxiety rating scale. Therefore, these studies might have affected the interpretation of the current meta-analysis.

Finally, to investigate the potential confounding effects of some clinical variables, we tried to conduct further exploratory subgroup analyses based on age. However, there were no significant findings from these subgroups. These results might be due to the smaller sample sizes after subgrouping.

### Limitations

This article had several limitations and the findings need to be considered with caution. First, our participant population is too heterogeneous because of our broad inclusion criteria, which might be true if considering current *Diagnostic and Statistical Manual of Mental Disorders* or *International Classification of Diseases* diagnostic systems. However, the novel Research Domain Criteria consider anxiety to be one of the major domains in Negative Valence Systems. Trials should be conducted in populations in which anxiety is the main symptom irrespective of the presence or absence of diagnosis of anxiety disorder. Second, because of the limited number of recruited studies and their modest sample sizes, the results should not be extrapolated without careful consideration. Third, the significant heterogeneity among the included studies (Cochran Q, 178.820; *df*, 18; *I*^2^, 89.934%; *P* < .001) with potential influence by some outlier studies, such as the studies by Sohrabi et al^56^ and Yehuda et al,^61^ would be another major concern. Therefore, clinicians should pay attention to this aspect when applying the results of the current meta-analysis to clinical practice, particularly when considering the subgroups of these 2 studies (ie, subgroups with specific clinical diagnoses, with <2000 mg/d, with EPA <60%, and with placebo-controlled trials).

## Conclusions

This systematic review and meta-analysis of clinical trials conducted on participants with clinical anxiety symptoms provides the first meta-analytic evidence, to our knowledge, that omega-3 PUFA treatment may be associated with anxiety reduction, which might not only be due to a potential placebo effect, but also from some associations of treatment with reduced anxiety symptoms. The beneficial anxiolytic effects of omega-3 PUFAs might be stronger in participants with specific clinical diagnoses than in those without specific clinical conditions. Larger and well-designed clinical trials should be performed with high-dose omega-3 PUFAs, provided as monotherapy and as adjunctive treatment to standard therapy.

## Notes

Supplement. **eAppendix.** Excluded Studies and Reasons **eTable.** Study Design and Jadad Scores of Recruited Studies **eFigure 1.** Whole Flowchart of Current Meta-Analysis **eFigure 2.** Funnel Plot of Changes in Anxiety Symptoms in Patients With and Without n-3 PUFA Treatment **eFigure 3.** Subgroup MA of Anxiolytic Effect Based Upon Placebo Controlled or Non–Placebo Controlled Design
