# Reanalysis After Retraction of Included Article

**DOI:** 10.1001/jamanetworkopen.2023.10754

**Published:** 2023-04-12

In the Original Investigation titled “Association of Use of Omega-3 Polyunsaturated Fatty Acids With Changes in Severity of Anxiety Symptoms: A Systematic Review and Meta-analysis,”^1^ published September 14, 2018, 1 of the articles included in analysis has since been retracted. A Comment was posted with the article on March 9, 2023, that describes a new analysis excluding the retracted article.^1^
