# Supplementary material for: Association of Use of Omega-3 Polyunsaturated Fatty Acids With Changes in Severity of Anxiety Symptoms: A Systematic Review and Meta-analysis
Source: JAMA Netw Open. 2018 Sep 14;1(5):e182327. doi: 10.1001/jamanetworkopen.2018.2327 (PMC6324500; doi:10.1001/jamanetworkopen.2018.2327)
Supplement: Supplement. — eAppendix. Excluded Studies and Reasons eTable. Study Design and Jadad Scores of Recruited Studies eFigure 1. Whole Flowchart of Current Meta-Analysis eFigure 2. Funnel Plot of Changes in Anxiety Symptoms in Patients With and Without n-3 PUFA Treatment eFigure 3. Subgroup MA of Anxiolytic Effect Based Upon Placebo Controlled or Non–Placebo Controlled Design [file jamanetwopen-1-e182327-s001.pdf]

Supplementary Online Content

Su K-P, Tseng P-T, Lin P-Y, et al. Association of use of omega-3 polyunsaturated fatty acids with changes in severity of anxiety symptoms: a systematic review and meta-analysis. *JAMA Netw Open*. 2018;1(5):e182327. doi:10.1001/jamanetworkopen.2018.2327

eAppendix. Excluded Studies and Reasons
eTable. Study Design and Jadad Scores of Recruited Studies
eFigure 1. Whole Flowchart of Current Meta-Analysis
eFigure 2. Funnel Plot of Changes in Anxiety Symptoms in Patients With and Without n-3 PUFA Treatment
eFigure 3. Subgroup MA of Anxiolytic Effect Based Upon Placebo Controlled or Non–Placebo Controlled Design

This supplementary material has been provided by the authors to give readers additional information about their work.

© 2018 Su K-P et al. *JAMA Network Open*.

eAppendix. Excluded Studies and Reasons

<u>Case report (n=2)</u>

1. Del Casale A, Serata D, Rapinesi C, et al. Psychosis risk syndrome comorbid with panic attack disorder in a cannabis-abusing patient affected by Arnold-Chiari malformation type I. *General hospital psychiatry.* 2012;34(6):702 e705-707.
2. Hou YC, Lai CH. Enhancing therapeutic effects after augmentation of omega-3 fatty acid with long-term antidepressant therapy in a chronic case of panic disorder. *Psychiatry Clin Neurosci.* 2016;70(1):72-73.

<u>Lack of adequate control (n=15)</u>

3. Trebaticka J, Hradecna Z, Bohmer F, et al. Emulsified omega-3 fatty-acids modulate the symptoms of depressive disorder in children and adolescents: a pilot study. *Child Adolesc Psychiatry Ment Health.* 2017;11:30.
4. Matsuoka Y, Nishi D, Yonemoto N, Hamazaki K, Hashimoto K, Hamazaki T. Omega-3 fatty acids for secondary prevention of posttraumatic stress disorder after accidental injury: an open-label pilot study. *J Clin Psychopharmacol.* 2010;30(2):217-219.
5. Matsuoka Y, Nishi D, Yonemoto N, Hamazaki K, Hamazaki T, Hashimoto K. Potential role of brain-derived neurotrophic factor in omega-3 Fatty Acid supplementation to prevent posttraumatic distress after accidental injury: an open-label pilot study. *Psychother Psychosom.* 2011;80(5):310-312.
6. Matsumura K, Noguchi H, Nishi D, Matsuoka Y. The effect of omega-3 fatty acids on psychophysiological assessment for the secondary prevention of posttraumatic stress disorder: an open-label pilot study. *Glob J Health Sci.* 2011;4(1):3-9.
7. Su KP, Yang HT, Chang JP, et al. Eicosapentaenoic and docosahexaenoic acids have different effects on peripheral phospholipase A2 gene expressions in acute depressed patients. *Progress in neuro-psychopharmacology & biological psychiatry.* 2018;80(Pt C):227-233.
8. Gananca L, Galfalvy HC, Oquendo MA, et al. Lipid correlates of antidepressant response to omega-3 polyunsaturated fatty acid supplementation: A pilot study. *Prostaglandins Leukot Essent Fatty Acids.* 2017;119:38-44.
9. Woo J, Couturier J, Pindiprolu B, et al. Acceptability and tolerability of omega-3 fatty acids as adjunctive treatment for children and adolescents with eating disorders. *Eat Disord.* 2017;25(2):114-121.

© 2018 Su K-P et al. *JAMA Network Open.*

10. Chong MF, Ong YL, Calder PC, et al. Long-chain polyunsaturated fatty acid status during pregnancy and maternal mental health in pregnancy and the postpartum period: results from the GUSTO study. *The Journal of clinical psychiatry.* 2015;76(7):e848-856.
11. Daley C, Patterson A, Sibbritt D, MacDonald-Wicks L. Unsaturated fat intakes and mental health outcomes in young women from the Australian Longitudinal Study on Women's Heath. *Public health nutrition.* 2015;18(3):546-553.
12. Giles GE, Mahoney CR, Urry HL, Brunye TT, Taylor HA, Kanarek RB. Omega-3 fatty acids and stress-induced changes to mood and cognition in healthy individuals. *Pharmacology, biochemistry, and behavior.* 2015;132:10-19.
13. Vaz Jdos S, Kac G, Emmett P, Davis JM, Golding J, Hibbeln JR. Dietary patterns, n-3 fatty acids intake from seafood and high levels of anxiety symptoms during pregnancy: findings from the Avon Longitudinal Study of Parents and Children. *PloS one.* 2013;8(7):e67671.
14. Mischoulon D, Best-Popescu C, Laposata M, et al. A double-blind dose-finding pilot study of docosahexaenoic acid (DHA) for major depressive disorder. *European neuropsychopharmacology : the journal of the European College of Neuropsychopharmacology.* 2008;18(9):639-645.
15. Wozniak J, Biederman J, Mick E, et al. Omega-3 fatty acid monotherapy for pediatric bipolar disorder: a prospective open-label trial. *European neuropsychopharmacology : the journal of the European College of Neuropsychopharmacology.* 2007;17(6-7):440-447.
16. Marangell LB, Martinez JM, Zboyan HA, Chong H, Puryear LJ. Omega-3 fatty acids for the prevention of postpartum depression: negative data from a preliminary, open-label pilot study. *Depression and anxiety.* 2004;19(1):20-23.
17. Suzuki S, Akechi T, Kobayashi M, et al. Daily omega-3 fatty acid intake and depression in Japanese patients with newly diagnosed lung cancer. *Br J Cancer.* 2004;90(4):787-793.

Meta-analysis (n=2)

18. Mocking RJ, Harmsen I, Assies J, Koeter MW, Ruhe HG, Schene AH. Meta-analysis and meta-regression of omega-3 polyunsaturated fatty acid supplementation for major depressive disorder. *Transl Psychiatry.* 2016;6:e756.
19. Appleton KM, Sallis HM, Perry R, Ness AR, Churchill R. Omega-3 fatty acids for depression in adults. *The Cochrane database of systematic reviews.* 2015(11):CD004692.

© 2018 Su K-P et al. *JAMA Network Open*.

Not human clinical trial (n=5)

20. Wu A, Noble EE, Tyagi E, Ying Z, Zhuang Y, Gomez-Pinilla F. Curcumin boosts DHA in the brain: Implications for the prevention of anxiety disorders. *Biochim Biophys Acta.* 2015;1852(5):951-961.
21. Hakimian J, Minasyan A, Zhe-Ying L, et al. Specific behavioral and cellular adaptations induced by chronic morphine are reduced by dietary omega-3 polyunsaturated fatty acids. *PloS one.* 2017;12(4):e0175090.
22. Hryhorczuk C, Decarie-Spain L, Sharma S, et al. Saturated high-fat feeding independent of obesity alters hypothalamus-pituitary-adrenal axis function but not anxiety-like behaviour. *Psychoneuroendocrinology.* 2017;83:142-149.
23. Robertson RC, Seira Oriach C, Murphy K, et al. Omega-3 polyunsaturated fatty acids critically regulate behaviour and gut microbiota development in adolescence and adulthood. *Brain, behavior, and immunity.* 2017;59:21-37.
24. Bandaru SS, Lin K, Roming SL, Vellipuram R, Harney JP. Effects of PI3K inhibition and low docosahexaenoic acid on cognition and behavior. *Physiol Behav.* 2010;100(3):239-244.

Not human clinical trial in the part of anxiety study (n=1)

25. Hamazaki T, Sawazaki S, Nagasawa T, Nagao Y, Kanagawa Y, Yazawa K. Administration of docosahexaenoic acid influences behavior and plasma catecholamine levels at times of psychological stress. *Lipids.* 1999;34 Suppl:S33-37.

Not related to omega 3 supplement effect on anxiety (n=38)

26. Liu JJ, Galfalvy HC, Cooper TB, et al. Omega-3 polyunsaturated fatty acid (PUFA) status in major depressive disorder with comorbid anxiety disorders. *The Journal of clinical psychiatry.* 2013;74(7):732-738.
27. Jadoon A, Chiu CC, McDermott L, et al. Associations of polyunsaturated fatty acids with residual depression or anxiety in older people with major depression. *Journal of affective disorders.* 2012;136(3):918-925.
28. Lesperance F, Frasure-Smith N, St-Andre E, Turecki G, Lesperance P, Wisniewski SR. The efficacy of omega-3 supplementation for major depression: a randomized controlled trial. *The Journal of clinical psychiatry.* 2011;72(8):1054-1062.
29. Sanchez-Villegas A, Henriquez P, Figueiras A, Ortuno F, Lahortiga F, Martinez-

© 2018 Su K-P et al. *JAMA Network Open*.

Gonzalez MA. Long chain omega-3 fatty acids intake, fish consumption and mental disorders in the SUN cohort study. *European journal of nutrition.* 2007;46(6):337-346.

30. Green P, Hermesh H, Monselise A, Marom S, Presburger G, Weizman A. Red cell membrane omega-3 fatty acids are decreased in nondepressed patients with social anxiety disorder. *European neuropsychopharmacology : the journal of the European College of Neuropsychopharmacology.* 2006;16(2):107-113.
31. Matsuoka Y, Nishi D, Tanima Y, et al. Serum pro-BDNF/BDNF as a treatment biomarker for response to docosahexaenoic acid in traumatized people vulnerable to developing psychological distress: a randomized controlled trial. *Transl Psychiatry.* 2015;5:e596.
32. Matsumura K, Noguchi H, Nishi D, Hamazaki K, Hamazaki T, Matsuoka YJ. Effects of omega-3 polyunsaturated fatty acids on psychophysiological symptoms of post-traumatic stress disorder in accident survivors: A randomized, double-blind, placebo-controlled trial. *Journal of affective disorders.* 2016.
33. Emmerich T, Abdullah L, Crynen G, et al. Plasma Lipidomic Profiling in a Military Population of Mild Traumatic Brain Injury and Post-Traumatic Stress Disorder with Apolipoprotein E varepsilon4-Dependent Effect. *J Neurotrauma.* 2016;33(14):1331-1348.
34. Verly-Miguel MV, Farias DR, Pinto Tde J, Lepsch J, Nardi AE, Kac G. Serum docosahexaenoic acid (DHA) is inversely associated with anxiety disorders in early pregnancy. *Journal of anxiety disorders.* 2015;30:34-40.
35. Saunders EF, Reider A, Singh G, Gelenberg AJ, Rapoport SI. Low unesterified:esterified eicosapentaenoic acid (EPA) plasma concentration ratio is associated with bipolar disorder episodes, and omega-3 plasma concentrations are altered by treatment. *Bipolar disorders.* 2015;17(7):729-742.
36. Hamazaki K, Takamori A, Tsuchida A, et al. Dietary intake of fish and n-3 polyunsaturated fatty acids and risks of perinatal depression: The Japan Environment and Children's Study (JECS). *Journal of psychiatric research.* 2018;98:9-16.
37. Thesing CS, Bot M, Milaneschi Y, Giltay EJ, Penninx B. Omega-3 and omega-6 fatty acid levels in depressive and anxiety disorders. *Psychoneuroendocrinology.* 2018;87:53-62.
38. Bowler RM, Adams SW, Schwarzer R, et al. Validity of self-reported concentration and memory problems: Relationship with neuropsychological assessment and depression. *J Clin Exp Neuropsychol.* 2017;39(10):1026-1036.

© 2018 Su K-P et al. *JAMA Network Open*.

39. Parellada M, Llorente C, Calvo R, et al. Randomized trial of omega-3 for autism spectrum disorders: Effect on cell membrane composition and behavior. *European neuropsychopharmacology : the journal of the European College of Neuropsychopharmacology.* 2017;27(12):1319-1330.
40. Pawelczyk T, Piatkowska-Janko E, Bogorodzki P, et al. Omega-3 fatty acid supplementation may prevent loss of gray matter thickness in the left parieto-occipital cortex in first episode schizophrenia: A secondary outcome analysis of the OFFER randomized controlled study. *Schizophr Res.* 2017.
41. Song L, Yoshida S, Tanaka-Mizuno S, Ogawa Y, Furukawa TA, Kawakami K. Association between eicosapentaenoic acid (EPA) medication intake and new onset of depression among Japanese patients with hyperlipidemia: A 3-year follow-up study. *J Nutr Intermed Metab.* 2017;9:12-16.
42. Wilson PB, Madrigal LA. Associations among Omega-3 Fatty Acid Status, Anxiety, and Mental Toughness in Female Collegiate Athletes. *J Am Coll Nutr.* 2017;36(8):602-607.
43. Lotrich FE, Sears B, McNamara RK. Polyunsaturated fatty acids moderate the effect of poor sleep on depression risk. *Prostaglandins Leukot Essent Fatty Acids.* 2016;106:19-25.
44. Chalut-Carpentier A, Pataky Z, Golay A, Bobbioni-Harsch E. Involvement of dietary Fatty acids in multiple biological and psychological functions, in morbidly obese subjects. *Obesity surgery.* 2015;25(6):1031-1038.
45. Hansen AL, Olson G, Dahl L, et al. Reduced anxiety in forensic inpatients after a long-term intervention with Atlantic salmon. *Nutrients.* 2014;6(12):5405-5418.
46. Su KP, Lai HC, Yang HT, et al. Omega-3 fatty acids in the prevention of interferon-alpha-induced depression: results from a randomized, controlled trial. *Biological psychiatry.* 2014;76(7):559-566.
47. Barbadoro P, Annino I, Ponzio E, et al. Fish oil supplementation reduces cortisol basal levels and perceived stress: a randomized, placebo-controlled trial in abstinent alcoholics. *Mol Nutr Food Res.* 2013;57(6):1110-1114.
48. Parletta N, Cooper P, Gent DN, Petkov J, O'Dea K. Effects of fish oil supplementation on learning and behaviour of children from Australian Indigenous remote community schools: a randomised controlled trial. *Prostaglandins Leukot Essent Fatty Acids.* 2013;89(2-3):71-79.
49. Souied EH, Delcourt C, Querques G, et al. Oral docosahexaenoic acid in the prevention of exudative age-related macular degeneration: the Nutritional AMD Treatment 2 study. *Ophthalmology.* 2013;120(8):1619-1631.
© 2018 Su K-P et al. *JAMA Network Open*.

50. Young L, Kemper KJ. Integrative care for pediatric patients with pain. *Journal of alternative and complementary medicine.* 2013;19(7):627-632.
51. Beezhold BL, Johnston CS. Restriction of meat, fish, and poultry in omnivores improves mood: a pilot randomized controlled trial. *Nutr J.* 2012;11:9.
52. Lok A, Assies J, Koeter MW, et al. Sustained medically unexplained physical symptoms in euthymic patients with recurrent depression: predictive value for recurrence and associations with omega 3- and 6 fatty acids and 5-HTTLPR? *Journal of affective disorders.* 2012;136(3):604-611.
53. Manor I, Magen A, Keidar D, et al. The effect of phosphatidylserine containing Omega3 fatty-acids on attention-deficit hyperactivity disorder symptoms in children: a double-blind placebo-controlled trial, followed by an open-label extension. *European psychiatry : the journal of the Association of European Psychiatrists.* 2012;27(5):335-342.
54. Beezhold BL, Johnston CS, Daigle DR. Vegetarian diets are associated with healthy mood states: a cross-sectional study in seventh day adventist adults. *Nutr J.* 2010;9:26.
55. Nguemeni C, Delplanque B, Rovere C, et al. Dietary supplementation of alpha-linolenic acid in an enriched rapeseed oil diet protects from stroke. *Pharmacological research.* 2010;61(3):226-233.
56. Ramsden C, Gagnon C, Graciosa J, et al. Do omega-6 and trans fatty acids play a role in complex regional pain syndrome? A pilot study. *Pain medicine.* 2010;11(7):1115-1125.
57. Hibbeln JR, Davis JM. Considerations regarding neuropsychiatric nutritional requirements for intakes of omega-3 highly unsaturated fatty acids. *Prostaglandins Leukot Essent Fatty Acids.* 2009;81(2-3):179-186.
58. Appleton KM, Gunnell D, Peters TJ, Ness AR, Kessler D, Rogers PJ. No clear evidence of an association between plasma concentrations of n-3 long-chain polyunsaturated fatty acids and depressed mood in a non-clinical population. *Prostaglandins Leukot Essent Fatty Acids.* 2008;78(6):337-342.
59. Emsley R, Niehaus DJ, Oosthuizen PP, et al. Safety of the omega-3 fatty acid, eicosapentaenoic acid (EPA) in psychiatric patients: results from a randomized, placebo-controlled trial. *Psychiatry research.* 2008;161(3):284-291.
60. Appleton KM, Peters TJ, Hayward RC, et al. Depressed mood and n-3 polyunsaturated fatty acid intake from fish: non-linear or confounded association? *Soc Psychiatry Psychiatr Epidemiol.* 2007;42(2):100-104.
61. Nieminen LR, Makino KK, Mehta N, Virkkunen M, Kim HY, Hibbeln JR.
© 2018 Su K-P et al. *JAMA Network Open*.

Relationship between omega-3 fatty acids and plasma neuroactive steroids in alcoholism, depression and controls. *Prostaglandins Leukot Essent Fatty Acids.* 2006;75(4-5):309-314.
62. Hibbeln JR, Bissette G, Umhau JC, George DT. Omega-3 status and cerebrospinal fluid corticotrophin releasing hormone in perpetrators of domestic violence. *Biological psychiatry.* 2004;56(11):895-897.
63. Williams LL, Kiecolt-Glaser JK, Horrocks LA, Hillhouse JT, Glaser R. Quantitative association between altered plasma esterified omega-6 fatty acid proportions and psychological stress. *Prostaglandins Leukot Essent Fatty Acids.* 1992;47(2):165-170.

Not simply treat with omega-3 related product (n=3)

64. Han X, Eggett DL, Parker TL. Evaluation of the Health Benefits of a Multivitamin, Multimineral, Herbal, Essential Oil-Infused Supplement: A Pilot Trial. *J Diet Suppl.* 2018;15(2):153-160.
65. Jamilian M, Shojaei A, Samimi M, et al. The effects of omega-3 and vitamin E co-supplementation on parameters of mental health and gene expression related to insulin and inflammation in subjects with polycystic ovary syndrome. *Journal of affective disorders.* 2018;229:41-47.
66. Barbarich NC, McConaha CW, Halmi KA, et al. Use of nutritional supplements to increase the efficacy of fluoxetine in the treatment of anorexia nervosa. *Int J Eat Disord.* 2004;35(1):10-15.

Protocol but no result of studies (n=2)

67. Matsuoka Y, Nishi D, Nakaya N, et al. Attenuating posttraumatic distress with omega-3 polyunsaturated fatty acids among disaster medical assistance team members after the Great East Japan Earthquake: the APOP randomized controlled trial. *BMC psychiatry.* 2011;11:132.
68. Matsuoka Y, Nishi D, Yonemoto N, et al. Tachikawa project for prevention of posttraumatic stress disorder with polyunsaturated fatty acid (TPOP): study protocol for a randomized controlled trial. *BMC psychiatry.* 2013;13:8.

Review article (n=7)

69. Bozzatello P, Brignolo E, De Grandi E, Bellino S. Supplementation with Omega-3 Fatty Acids in Psychiatric Disorders: A Review of Literature Data. *J Clin Med.* 2016;5(8).

© 2018 Su K-P et al. *JAMA Network Open*.

70. Su KP, Matsuoka Y, Pae CU. Omega-3 Polyunsaturated Fatty Acids in Prevention of Mood and Anxiety Disorders. *Clin Psychopharmacol Neurosci.* 2015;13(2):129-137.
71. Ravindran AV, da Silva TL. Complementary and alternative therapies as add-on to pharmacotherapy for mood and anxiety disorders: a systematic review. *Journal of affective disorders.* 2013;150(3):707-719.
72. Galan-Arriero I, Serrano-Munoz D, Gomez-Soriano J, et al. The role of Omega-3 and Omega-9 fatty acids for the treatment of neuropathic pain after neurotrauma. *Biochim Biophys Acta.* 2017;1859(9 Pt B):1629-1635.
73. Nock TG, Chouinard-Watkins R, Plourde M. Carriers of an apolipoprotein E epsilon 4 allele are more vulnerable to a dietary deficiency in omega-3 fatty acids and cognitive decline. *Biochim Biophys Acta.* 2017;1862(10 Pt A):1068-1078.
74. Cardoso C, Afonso C, Bandarra NM. Dietary DHA and health: cognitive function ageing. *Nutrition research reviews.* 2016;29(2):281-294.
75. Ross BM. Omega-3 polyunsaturated fatty acids and anxiety disorders. *Prostaglandins Leukot Essent Fatty Acids.* 2009;81(5-6):309-312.

<u>The same sample source from Buydens-Branchey, L. (2008) (n=1)</u>

76. Buydens-Branchey L, Branchey M. n-3 polyunsaturated fatty acids decrease anxiety feelings in a population of substance abusers. *J Clin Psychopharmacol.* 2006;26(6):661-665.

<u>The same sample source from Nishi, D. (2013) (n=1)</u>

77. Nishi D, Koido Y, Nakaya N, et al. Fish oil for attenuating posttraumatic stress symptoms among rescue workers after the great east Japan earthquake: a randomized controlled trial. *Psychother Psychosom.* 2012;81(5):315-317.

<u>The same sample source from Matsuoka, Y. (2015) (n=1)</u>

78. Noguchi H, Nishi D, Matsumura K, Hamazaki K, Hamazaki T, Matsuoka YJ. Limited effect of omega-3 fatty acids on the quality of life in survivors of traumatic injury: A randomized, placebo-controlled trial. *Prostaglandins Leukot Essent Fatty Acids.* 2017;127:1-5.

<u>Database study but not clinical trials (n=1)</u>

79. Adams J, Sibbritt D, Lui CW, Broom A, Wardle J. {Omega}-3 fatty acid
© 2018 Su K-P et al. *JAMA Network Open*.

supplement use in the 45 and Up Study Cohort. *BMJ Open.* 2013;3(4).

No detailed information about changes of anxiety symptoms despite of our request of detailed data (n=6)

80. Witte AV, Kerti L, Hermannstadter HM, et al. Long-chain omega-3 fatty acids improve brain function and structure in older adults. *Cereb Cortex.* 2014;24(11):3059-3068.
81. Fontani G, Corradeschi F, Felici A, Alfatti F, Migliorini S, Lodi L. Cognitive and physiological effects of Omega-3 polyunsaturated fatty acid supplementation in healthy subjects. *European journal of clinical investigation.* 2005;35(11):691-699.
82. Jazayeri S, Keshavarz SA, Tehrani-Doost M, et al. Effects of eicosapentaenoic acid and fluoxetine on plasma cortisol, serum interleukin-1beta and interleukin-6 concentrations in patients with major depressive disorder. *Psychiatry research.* 2010;178(1):112-115.
83. Reed SD, Guthrie KA, Newton KM, et al. Menopausal quality of life: RCT of yoga, exercise, and omega-3 supplements. *Am J Obstet Gynecol.* 2014;210(3):244 e241-211.
84. Milte CM, Parletta N, Buckley JD, Coates AM, Young RM, Howe PR. Eicosapentaenoic and docosahexaenoic acids, cognition, and behavior in children with attention-deficit/hyperactivity disorder: a randomized controlled trial. *Nutrition.* 2012;28(6):670-677.
85. Ravi S, Khalili H, Abbasian L, Arbabi M, Ghaeli P. Effect of Omega-3 Fatty Acids on Depressive Symptoms in HIV-Positive Individuals: A Randomized, Placebo-Controlled Clinical Trial. *The Annals of pharmacotherapy.* 2016;50(10):797-807.

© 2018 Su K-P et al. *JAMA Network Open*.

eTable. Study Design and Jadad Scores of Recruited Studies

Author (year)	Study design	Diagnosis	Jadad Score			
			Random	Blind	Cohort	Total
Watanabe, N. (2018) [60]	Double-blind randomized placebo controlled	Junior nurses work in hospital	1	2	1	4
Cornu, C. (2017) [54]	Double-blind randomized placebo controlled	Children with ADHD	2	2	1	5
Matsuoka, Y. (2015) [51]	Double-blind randomized placebo controlled	Severely accidental injury	2	2	1	5
Bellino, S. (2014) [49]	Randomized controlled	Borderline personality disorders	1	0	1	2
Cohen, L.S. (2014) [50]	Double-blind randomized placebo controlled	Generally healthy subjects	2	2	1	5
Pomponi, M. (2014) [58]	Double-blind randomized placebo controlled	Parkinson's Disease	2	1	1	4
Widenhorn-Muller, K. (2014) [59]	Double-blind randomized placebo controlled	Children with ADHD	2	1	1	4
Haberka, M. (2013) [35]	Single-blind randomized controlled	Acute myocardial infarction	2	0	1	3
Nishi, D. (2013) [61]	Single-blind randomized controlled	Disaster-related trauma	2	0	1	3

© 2018 Su K-P et al. *JAMA Network Open*.

Sauder, K.A. (2013) [52]	Double-blind randomized placebo control cross-over	Healthy, nonsmoking men and postmenopausal women with moderate hypertriglyceridemia	1	1	1	3
Sohrabi, N. (2013) [53]	Double-blind randomized placebo controlled	Healthy women with premenstrual syndrome	1	2	1	4
Gabbay, V. (2012) [48]	Double-blind randomized placebo controlled	Tourette syndrome	1	2	1	4
Kiecolt-Glaser, J.K. (2011) [36]	Double-blind randomized placebo controlled	Generally healthy subjects	2	1	1	4
Buydens-Branchey, L. (2008) [34]	Double-blind randomized placebo controlled	Substance abuse	2	2	1	5
Freund-Levi, Y. (2008) [55]	Double-blind randomized placebo controlled	Alzheimer's disease	1	1	1	3
Rogers, P.J. (2008) [47]	Double-blind randomized placebo controlled	Mild to severe depression	1	2	1	4
van de Rest, O. (2008) [57]	Double-blind randomized placebo controlled	Elderly volunteers	2	2	1	5
Yehuda, S. (2005) [56]	Case-placebo control trial	Undergraduate college students with test anxiety	0	1	1	2
Fux, M. (2004) [33]	Double-blind randomized placebo controlled	Obsessive-compulsive disorder	1	1	1	3

© 2018 Su K-P et al. *JAMA Network Open*.

eFigure 1. Whole Flowchart of Current Meta-Analysis

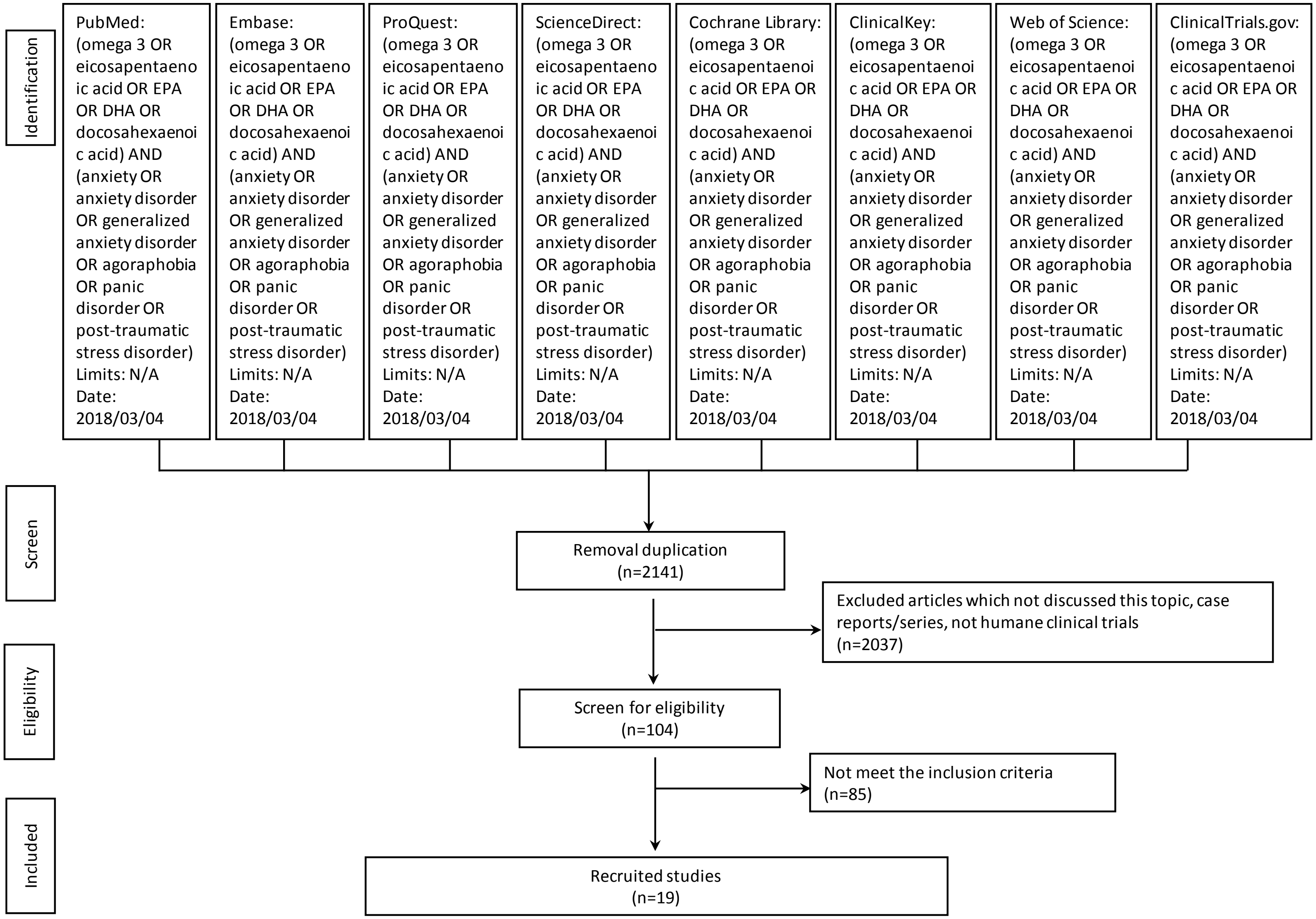

© 2018 Su K-P et al. *JAMA Network Open*.

eFigure 2. Funnel Plot of Changes in Anxiety Symptoms in Patients With and Without n-3 PUFA Treatment

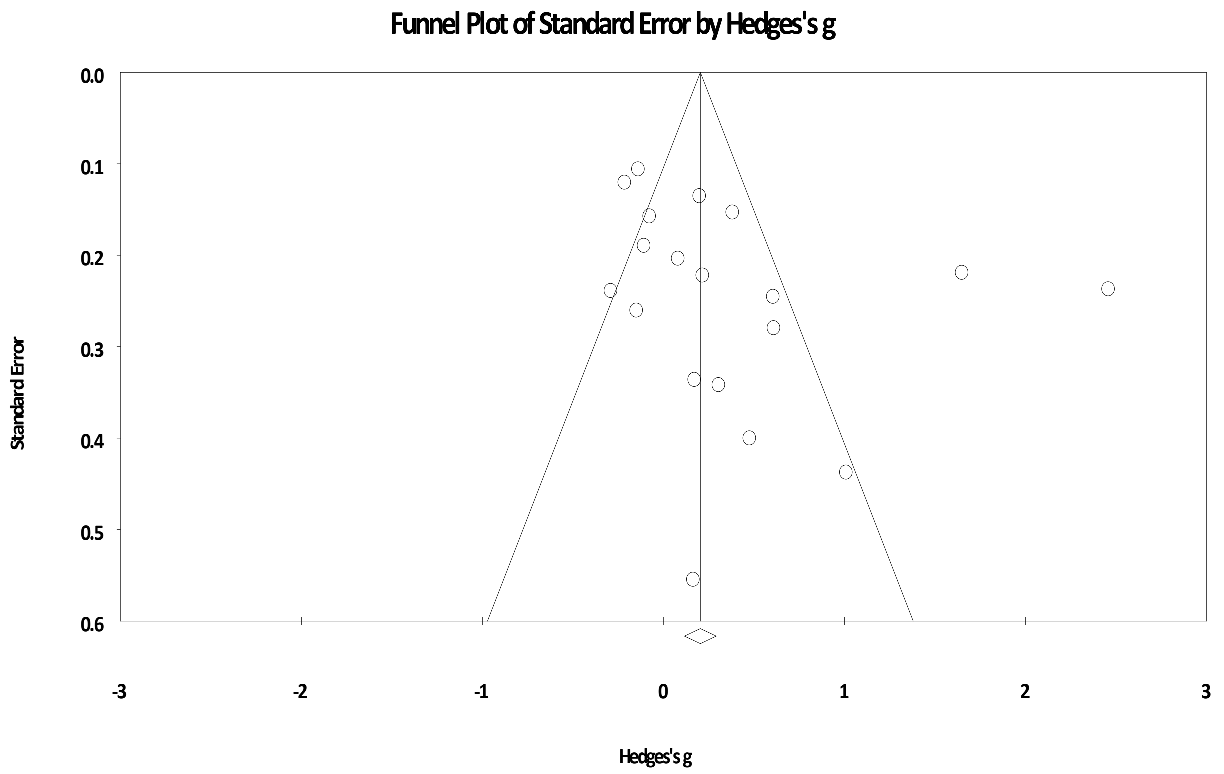

© 2018 Su K-P et al. *JAMA Network Open*.

eFigure 3. Subgroup MA of Anxiolytic Effect Based Upon Placebo Controlled or Non–Placebo Controlled Design

Compared to	Study name	Sample size: Omega 3	Sample size: control	Statistics for each study: Hedges' *g*	95% Low CI	95% Up CI	*p*-Value	Relative weight
Non-placebo	Bellino, S. (2014)[49]	18	16	0.172	-0.487	0.831	.61	13.78
	Nishi, D. (2013)[61]	86	86	0.382	0.082	0.683	.01	66.31
	Haberka, M. (2013)[35]	26	26	0.610	0.062	1.158	.03	19.91
	**Overall**			**0.399**	**0.154**	**0.643**	**<.01**	**100.00**
Placebo	Sauder, K.A. (2013)[52]	52	26	-0.290	-0.759	0.178	.22	6.41
	van de Rest, O. (2008)[57]	196	106	-0.214	-0.450	0.023	.08	7.05
	Freund-Levi, Y. (2008)[55]	36	24	-0.149	-0.659	0.362	.57	6.27
	Cohen, L.S. (2014)[50]	177	178	-0.139	-0.347	0.069	.19	7.10
	Matsuoka, Y. (2015)[51]	53	57	-0.107	-0.479	0.264	.57	6.71
	Cornu, C. (2017)[54]	79	81	-0.077	-0.385	0.232	.63	6.88
	Widenhorn-Muller, K. (2014)[59]	46	49	0.081	-0.318	0.481	.69	6.63
	Fux, M. (2004)[33]	6	5	0.165	-0.922	1.252	.77	4.18
	Rogers, P.J. (2008)[47]	109	109	0.200	-0.065	0.465	.14	6.99
	Watanabe, N. (2018)[60]	40	40	0.217	-0.219	0.652	.33	6.52
	Gabbay, V. (2012)[48]	17	16	0.306	-0.364	0.976	.37	5.69
	Pomponi, M. (2014)[58]	12	12	0.477	-0.308	1.261	.23	5.26
	Kiecolt-Glaser, J.K. (2011)[36]	34	34	0.607	0.126	1.088	.01	6.37
	Buydens-Branchey, L. (2008)[34]	11	11	1.010	0.153	1.868	.02	4.98
	Yehuda, S. (2005)[56]	88	38	1.650	1.220	2.079	<.001	6.54
	Sohrabi, N. (2013)[53]	63	61	2.459	1.994	2.923	<.001	6.42
	**Overall**			**0.372**	**0.032**	**0.712**	**.03**	**100.00**

Hedges' *g* and 95% CI

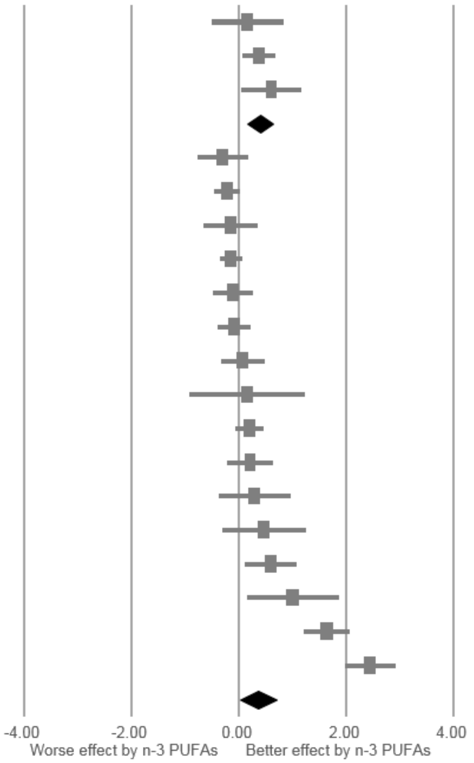

Subgroup meta-analysis of the anxiolytic effect by n-3 PUFAs based upon placebo-control or non-placebo-control. There was significantly better improvement in anxiety symptoms in patients treated with n-3 PUFAs compared with controls in subgroups of both placebo controls (k = 16, Hedges' g = 0.372, 95% CI = 0.032 to 0.712, p = 0.032) and non-placebo-control (k = 3, Hedges' g = 0.399, 95% CI = 0.154 to 0.643, p = 0.001)

© 2018 Su K-P et al. *JAMA Network Open*.
